# Antifungal Potential of Capsaicinoids and Capsinoids from the *Capsicum* Genus for the Safeguarding of Agrifood Production: Advantages and Limitations for Environmental Health

**DOI:** 10.3390/microorganisms10122387

**Published:** 2022-11-30

**Authors:** Jéssica Costa, Marcela Sepúlveda, Víctor Gallardo, Yasna Cayún, Christian Santander, Antonieta Ruíz, Marjorie Reyes, Carla Santos, Pablo Cornejo, Nelson Lima, Cledir Santos

**Affiliations:** 1 Departamento de Biologia, Instituto de Ciências Biológicas-ICB, Universidade Federal do Amazonas, Av. Rodrigo Otávio Jordão Ramos 3000, Bloco 01, Manaus 69077-000, AM, Brazil; 2 Department of Chemical Science and Natural Resources, Universidad de La Frontera, Temuco 4811-230, Chile; 3 Programa de Doctorado en Ciencias de Recursos Naturales, Universidad de La Frontera, Temuco 4811-230, Chile; 4 Environmental Engineering and Biotechnology Group, Faculty of Environmental Science and EULA-Chile Center, Universidad de Concepción, Concepción 4070-411, Chile; 5 CEB-Centre of Biological Engineering, Micoteca da Universidade do Minho (MUM), University of Minho, Campus de Gualtar, 4710-057 Braga, Portugal; 6 LABBELS (Associate Laboratory, Braga/Guimarães), University of Minho, Campus de Gualtar, 4710-057 Braga, Portugal; 7 Escuela de Agronomía, Facultad de Ciencias Agronómicas y de los Alimentos, Pontificia Universidad Católica de Valparaíso, Quillota 2260-000, Chile

**Keywords:** red pepper, capsaicinoids, pungency, plant defence, biofungicides, secondary metabolites

## Abstract

Opportunistic pathogenic fungi arise in agricultural crops as well as in surrounding human daily life. The recent increase in antifungal-resistant strains has created the need for new effective antifungals, particularly those based on plant secondary metabolites, such as capsaicinoids and capsinoids produced by *Capsicum* species. The use of such natural compounds is well-aligned with the One Health approach, which tries to find an equilibrium among people, animals, and the environment. Considering this, the main objective of the present work is to review the antifungal potential of capsaicinoids and capsinoids, and to evaluate the environmental and health impacts of biofungicides based on these compounds. Overall, capsaicinoids and their analogues can be used to control pathogenic fungi growth in plant crops, as eco-friendly alternatives to pest management, and assist in the conservation and long-term storage of agrifood products. Their application in different stages of the agricultural and food production chains improves food safety, nutritional value, and overcomes antimicrobial resistance, with a lower associated risk to humans, animals, and the environment than that of synthetic fungicides and pesticides. Nevertheless, research on the effect of these compounds on bee-like beneficial insects and the development of new preservatives and packaging materials is still necessary.

## 1. Introduction

### 1.1. Synthetic Fungicides and the One Health Concept

According to the World Health Organisation (WHO), the One Health concept is “an approach to designing and implementing programmes, policies, legislation, and research in which multiple sectors communicate and work together to achieve better public health outcomes” [1]. This is not a new concept, but it has achieved more visibility in recent years. Targets such as food safety, control of zoonosis, insect pests, and combating both microbial pathogenicity and antimicrobial resistance are well within the scope of the One Health concept. This means that the One Health concept is an approach that recognises the health of people, flora, and fauna are closely connected through a single shared environment [1].

The Food and Agriculture Organisation of the United Nations projects that the world population will reach 9.7 billion people by 2050, and 10.4 billion by the end of the century [2]. The world needs technologies that safeguard food and environmental security for this growing population scenario [2]. In this context, fungal diseases, particularly those affecting crop production, are a major threat. The estimated annual crop losses range from 20 to 40% worldwide, which translates to a yearly cost valued up to USD 220 billion on a global scale [3] and represents a risk to global food security and a threat to human wellbeing. Furthermore, an additional level of complexity is to be considered due to the emergence of antifungal resistance (AFR) developed by some fungal taxa. AFR is a global issue and has been a growing threat for many years, affecting all kinds of environments, including crops, food, animals, and people [4]. Such problems increase morbidity and mortality in humans and animals, causing an operational and economic burden on healthcare systems [5].

In 2020, fungicides accounted for more than half (56.8%) of products belonging to the “fungicides and bactericides” category sold in the European Union [6]. Fungicides used to control phytopathogens share the target activity with antimycotics used in livestock production and human clinical care. This represents a latent risk of the use of synthetic fungicides in the development of fungicide-resistant pathogens in agricultural practices [7]. In addition, this could lead to an increase in the emergence of human pathogenic fungi, the threat of which was previously restricted to agricultural crops.

The above statements have been corroborated by previous studies [7,8,9,10]. A specific example is the case of azoles, which have been the most widely used synthetic fungicide class for more than four decades, helping to control fungal plant diseases. Currently, more than 25 different synthetic azoles have been developed for the control of diseases in crops [11,12]. Azole fungicides are also frequently present in a myriad of personal care products, such as hair shampoo, soap, toothpaste, face and body cream, and shower gel, among others [12]. The indiscriminate use of synthetic fungicides in human healthcare and wellbeing, as well as in agricultural production, could strongly impact human, animal, and environmental health, being a key point for the development of AFR, which leads to a vicious cycle.

Although the environmental impact of synthetic fungicides has not often been assessed by analytical methods, there is evidence that farmers, their families, and those living adjacent to farming areas may face long-term health issues associated with the use of such xenobiotic compounds [13,14]. Synthetic fungicides also present a high level of toxicity to the surrounding animal communities. An example of this is the effect of synthetic fungicides on the health of a variety of both honeybee adults and larvae. Their ability to fly and their physiological development has been shown to be affected [15]. The different synthetic fungicide effects on environmental health reflect the pressing need for research and the development of biocompounds with at least similar potential to synthetic fungicides; however, with less environmental impact.

### 1.2. Capsicum spp. as a Source of Compounds with Natural Antifungal Activity

The *Capsicum* L. genus is one of the oldest cultivated plants in America, having been spread and established in many subtropical and tropical regions of the world [16]. From its outset, *Capsicum* spp. was used by American natives as a condiment and as medicine [17,18]. It belongs to the Solanaceae family, which also includes tomatoes (*Solanum lycopersicon* L.), potatoes (*Solanum tuberosum* L.), eggplants (*Solanum melongena* L.), and tobacco (*Nicotiana tabacum* L.) [19].

The *Capsicum* genus consists of up to 42 species of pepper, where *C. annuum*, *C. baccatum*, *C. frutescens*, *C. chinense*, and *C. pubescens* are the most commonly produced. Overall, *Capsicum* peppers are divided into two groups, pungent and non-pungent, also called hot and sweet peppers [20]. The pungency of *Capsicum* cultivars is closely related to the concentration of capsaicinoid molecules present in the pepper fruit [21]. The pungency index of peppers has been expressed in Scoville Heat Units (SHUs), which represent the number of dilutions in water required for a sample to lose its pungency sensation [22].

The pungency classification levels on the Scoville scale are: non-pungent (0–700 SHU), mildly pungent (700–3000 SHU), moderately pungent (3000–25,000 SHU), highly pungent (25,000–80,000 SHU), and very highly pungent (>80,000 SHU). For instance, the pure capsaicin score is about 16,000,000 units, while Carolina Reaper, a highly pungent pepper cultivar, reaches up to 2,200,000 SHUs [23].

Capsaicinoids are phenolic alkaloid compounds which include capsaicin (CAP) and dihydrocapsaicin (DHC), normally present as major components, and homocapsaicin (h-CAP), nordihydrocapsaicin (n-DHC), homo-dihydrocapsaicin (h-DHC), and nonivamide, that are generally present in trace amounts [24,25]. Together, CAP and DHC represent approximately 91% of total capsaicinoids in pepper fruits of *Capsicum*, with CAP and DHC accounting for c.a. 69 and 22%, respectively, in most of the pungent varieties of *Capsicum* spp. In addition, n-DHC represents c.a. 7% of the total capsaicinoids, while h-DHC and h-CAP represent c.a. 2% (1% each) of the total capsaicinoids amount in fruits of *Capsicum* spp. [26,27,28,29,30].

These secondary metabolites have been determined as part of the host plant’s defence mechanisms and play a key role as deterrents for herbivores and pathogenic fungi. However, the plant−fungi interspecies crosstalk and fungi-plant microbiota crosstalk are complex communication networks that can be affected by intrinsic and extrinsic factors [31,32,33]. The barriers established by the fruits’ bioactive metabolites can be deflected or defeated either by a selected group of fungi previously equipped to overcome capsaicinoid defence mechanisms or by a process of plant-fungus coevolution [32].

**Figure 1:**
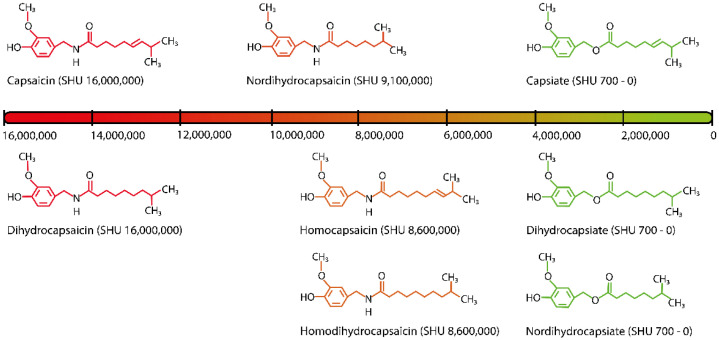
Scoville scale and molecular structure for the main capsaicinoids and capsinoids produced by species of *Capsicum* genus.

The capsaicinoid analogue group named “capsinoids” has been mainly, but not exclusively, identified in low-pungency *Capsicum* cultivars [34,35]. The capsinoids group encompasses capsiate, dihydrocapsiate, and nordihydrocapsiate. Aside from the pungency index, the main difference between these groups relies on their molecular structures: capsaicinoids have amide bonds, whereas capsinoids have ester bonds (Figure 1) [34,36]. Other non-pungent capsaicinoid analogues produced in *Capsicum* fruits, called “capsiconinoids” (e.g., capsiconiate and dihydrocapsiconiate), have also been identified. However, little information is available regarding this residual secondary metabolite group of the *Capsicum* genus [35,37].

The biosynthesis of capsaicinoids and capsinoids is genetically controlled, but it is also dependent on environmental conditions, plant genotype or cultivar, and fruiting and maturation stages [38,39]. Nevertheless, the ecological role of capsinoids and capsiconinoids remains poorly understood. Capsaicinoids have been shown to be remarkably valuable for human wellbeing. Due to the analgesia, anticancer, anti-inflammatory, antioxidative, anti-obesity, anti-virulence, and antimicrobial activities of capsaicinoid molecules, several studies have focused on their use in the development of cosmetic and pharmaceutical products [40,41,42,43]. In addition, the capsinoids group has similar medical and biotechnological applications with the added benefit of being less irritating and more palatable due to their low pungency.

Opportunistic pathogenic fungi arise in agricultural crops, as well as in surrounding human daily life. The increase in antifungal-resistant strains has prompted interest in new effective antifungal compounds based on plant-derived secondary metabolites. The effective management of fungal diseases, through bioactive compounds, can replace or minimise the use of synthetic antifungals, meeting the demands of end consumers for eco-friendly products, strengthening the One Health values. Therefore, One Health is a pivotal strategy for tackling pathogenic fungal strains whilst catering to all aspects of health care for humans, animals, and the environment [44]. Considering this, the main aim of this work is to review the antifungal potential of capsaicinoids and capsinoids present in different varieties of *Capsicum*, and to evaluate the impact of biofungicides produced from these natural compounds on agrifood production, as well as their advantages and limitations regarding environmental health.

## 2. Materials and Methods

The literature review was performed based on an analysis of scientific data published concerning the antifungal potential of molecules belonging to the classes of capsaicinoids and capsinoids present in different varieties of *Capsicum* genus; the impact of biofungicides produced with capsaicinoids and capsinoids on agrifood production; and their advantages and limitations regarding environmental health. The fragmented information was compiled and tabulated, allowing for gap analysis and identification of which problems need to be investigated in this field.

The concept map for the current review began with an analysis of capsaicinoids and capsinoids of *Capsicum* pepper and the key factors that affect its biosynthesis. Then, key information about the antifungal activity of these compounds was addressed, as well as considerations as to how the processing of *Capsicum* products from the field to post-harvest could affect the stability and concentration of capsaicinoids and capsinoids.

All scientific literature was taken into consideration with special attention paid to publications in the last decade (2012–2022), which represents more than 64% of the references used (101 out of 156 references used).

## 3. Characteristics of Capsaicinoids and Capsinoids

Capsaicinoids and capsinoids represent two classes of chemical compounds that are secondary metabolites exclusively produced by species of the *Capsicum* genus [20,30,34,35,45,46]. The biosynthesis of capsaicinoids and capsinoids occurs in the placenta of the *Capsicum* pods, being accumulated in vesicles of placenta epidermal cells and excreted on seeds and on the pericarp [24]. Furthermore, in highly pungent or ‘super-hot’ peppers, capsaicinoids can also be synthesised in vesicles on the inner surface of the pericarp [47,48], suggesting that capsaicinoids are part of the host plant’s defence compounds, providing protection against herbivory and microbial infections [30,49,50].

Regarding their chemical structure, capsaicinoids contain an amide bond, which is responsible for their pungency [51], instead of the ester bond found in capsinoids (Figure 1). In addition, the chemical backbone of capsaicinoid molecules is composed of a fatty acid amide linked to vanillylamine moiety; while that of capsinoids molecules contains a fatty acid ester bonded to vanillyl alcohol (Figure 1).

CAP ((*E*)-N-[(4-hydroxy-3-methoxyphenyl) methyl]-8-methylnon-6-enamide) has the molecular formula C_18_H_27_NO_3_ and a molecular mass of 305.40 g/mol. It is obtained from pepper fruits of the *Capsicum* genus as a crystalline, colourless, and odourless compound. The CAP molecule has a melting point of 62 to 65 °C; it is not soluble in water but presents relative solubility in ethanol, dimethyl sulfoxide, dimethyl formamide, acetone, and fatty oils. The solubility of capsaicin in these solvents is at least 30 mg/mL. The *trans* isomer of CAP is the most stable one and is the naturally occurring form of capsaicin, while the *cis* isomer is the less stable arrangement of the molecule [30,52,53,54].

DHC (*N*-[(4-hydroxy-3-methoxyphenyl)methyl]-8-methylnonanamide) has the molecular formula C_18_H_29_NO_3_ and a molecular mass of 307.43 g/mol. It is a capsaicinoid analogue and congener of capsaicin. Like CAP, DHC is an irritant compound with similar pungency to capsaicin (Figure 1). DHC is obtained from fruits of *Capsicum* spp. as a lipophilic, colourless, odourless, crystalline to waxy compound, and has a melting point of 65.5 to 65.8 °C. It presents a solubility of 14 mg/mL in water and is relatively soluble in chloroform, dimethyl sulfoxide, or 100% ethanol [30,54].

The remaining three major capsaicinoid compounds found in *Capsicum* spp. are n-DHC, h-DHC, and h-CAP, altogether forming no more than c.a. 9% of the total capsaicinoids in fruits of *Capsicum* spp. n-DHC (*N*-[(4-hydroxy-3-methoxyphenyl)methyl]-7-methylnonanamide) has the molecular formula C_17_H_27_NO_3_ and a molecular mass of 293.40 g/mol. It presents a melting point of 60 to 61 °C and has moderate solubility in chloroform, dichloromethane, ethyl acetate, dimethyl sulfoxide, and acetone [55].

h-DHC and h-CAP are capsaicinoids occurring in smallest abundance in pepper fruits. h-DHC (*N*-[(4-hydroxy-3-methoxyphenyl)methyl]-9-methyldecanamide) has the molecular formula C_19_H_31_NO_3_ and a molecular mass of 321.46 g/mol. The h-DHC melting point is 70 to 71 °C, and this molecule is soluble in dimethyl sulfoxide (100 mg/mL). h-CAP ((*E*)-*N*-[(4-hydroxy-3-methoxyphenyl)methyl]-9-methyldec-7-enamide) has the molecular formula C_19_H_29_NO_3_ and a molecular mass of 319.43 g/mol. The h-CAP melting point is 64.5 to 65.5 °C, and this compound is soluble in dimethyl sulfoxide [30,55]. Homohydrocapsaicin and noviamide are also capsaicinoid molecules found in a residual amount in pepper fruits of the *Capsicum* genus [56].

Capsinoids were first reported by Yazawa et al. [34] in the non-pungent pepper cultivar CH-19 Sweet. To date, three capsinoids have been described in pepper fruits: capsiate, dihydrocapsiate, and nordihydrocapsiate. Capsiate (4-hydroxy-3-methoxyphenyl)methyl (*E*)-8-methylnon-6-enoate) has the molecular formula C_18_H_26_O_4_ and a molecular mass of 306.18 g/mol. It is the major compound among capsinoids found in the *Capsicum* pepper [35].

Dihydrocapsiate (4-hydroxy-3-methoxyphenyl)methyl 8-methylnonanoate) has the molecular formula C_18_H_28_O_4_ and a molecular mass of 308.41 g/mol. It is the second capsinoid compound in terms of its natural abundance in pepper fruits of *Capsicum* spp. Finally, nordihydrocapsiate (4-hydroxy-3-methoxyphenyl)methyl 7-methyloctanoate) has the molecular formula C_17_H_26_O_4_ and a molecular mass of 294.18 g/mol. It is the less abundant compound of the capsinoid family, naturally occurring in the pepper fruits of *Capsicum* spp. [35].

According to previous studies, capsinoids present similar physiological effects in plants as those observed for capsaicinoid molecules [55,57]. The gap in pungency in capsinoids molecules could be an advantage when using these compounds in some biotechnological processes. In fact, pungency is a relevant chemical characteristic for the application of capsaicinoids in food, beverages, and pharmacological processes. However, the use of irritant compounds can represent a limitation in the production of some cosmetics or even during the processing of pepper pods at laboratorial, semi-industrial, or industrial levels during the development of biotechnology-based products. Based on our research conducted at both laboratory and semi-industrial levels, we have observed the challenges of manipulating fruits of pungent varieties of different *Capsicum* species [18,20,58,59].

Capsiconinoid molecules such as capsiconiate and dihydrocapsiconiate are non-pungent capsaicin-related molecules that are found in residual amounts in fruits of different varieties in the *Capsicum* genus [35,60]. The occurrence of both capsinoids and capsiconinoids in low and very low concentrations, respectively, in pepper fruits of *Capsicum* spp. can be a limitation for their use in the form of pure natural extracts. However, such compounds can have synergistic activity, together with major capsaicinoid compounds, regarding their antimicrobial potential.

## 4. External Factors That Affect Capsaicinoids and Capsinoids

**Figure 2:**
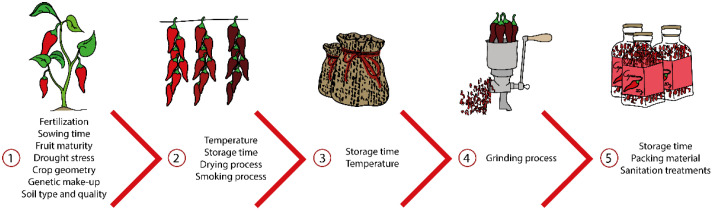
Extrinsic and intrinsic factors that can affect capsaicinoids and capsinoids biosynthesis and degradation throughout the *Capsicum* production chain.

The biosynthesis and accumulation of Capsaicinoids and capsinoids can be affected by *Capsicum* genotype, agronomic parameters (soil type and quality, sowing time, fertilization, fruit maturity, crop geometry), environmental factors (temperature, drought stresses), and postharvest processing (drying and/or smoking processes, storage conditions, sanitation treatments, and packing) (Figure 2) [61,62,63,64]. 

On the field, soil type and quality strongly affect *Capsicum* physiology [65,66,67]. Das et al. [66] reported that capsaicin biosynthesis of *C. frutescens* cv. Nodaria and *C. annuum* cv. Balijur were superior in alluvial soil than in lateritic soil. According to the author, soil organic carbon (SOC), fulvic acid carbon (FAC), humic acid carbon (HAC), total nitrogen, and microbial biomass-enriched alluvial quality buster capsaicin biosynthesis. In addition, good fertilisation management influences capsaicinoid accumulation on fruit (Figure 2). Mineral supplementation (N-P-K) and organic inputs (e.g., vermicompost) on soil can promote plant growth and the pungency of *Capsicum* [67,68]. However, each landrace of *Capsicum* shows a different level of adaptability to each soil type and fertiliser approach, showing that the genetic make-up of *Capsicum* landrace plays a key role in capsaicinoids and capsinoids accumulation. Indeed, the content of capsaicinoids and capsinoids in *Capsicum* cultivars can be more or less stable depending on different environments (Figure 2) [67,68]. Gurung et al. [69] analysed the yield and stability of capsaicinoid content in *Capsicum* spp. cultivated in six different environments. According to the authors, the cultivar Dallay khorsaney had high capsaicin, dihydrocapsaicin, and total capsaicinoids content, yet was very sensitive to environmental changes. In contrast, the cultivar KKU-P-11003 was more stable and suitable for diverse environments. Mahmood et al. [63] reported that stress suffered during *Capsicum* pod formation may restrict capsaicin synthase activity, affecting pepper pungency in the case of both pungent pepper varieties Pusajuala and Ghotki (*Capsicum annuum*). Overall, the content of capsaicinoid and capsinoid compounds in the *Capsicum* plant begins to accumulate 10–20 days after flowering (DAF) and continues to accumulate until 30–50 DAF [70,71]. Some studies have suggested that capsaicinoids and capsinoids content continues to increase until the fruit ripens [72,73,74]; while other studies have demonstrated a sharp decrease during the last stages of development [62,75,76]. Other agronomic parameters such as sowing time, fruit node position, and crop geometry are relevant to *Capsicum* fruit productivity, and consequently, could affect the plant metabolite composition. Up to now, their roles in the accumulation and biosynthesis of capsaicinoids and capsinoids have been poorly evaluated [47,77,78].

*Capsicum* fruits undergo several post-harvest procedures, such as smoking and drying processes, following traditional methods (e.g., smoking firewood, long-term sun-dried) or industrial methods (e.g., short-term heat treatment) which can differently affect their physical, chemical, and nutritional components.

Topuz et al. [79] analysed the influence of drying methods on the capsaicinoid composition of Jalapeno (*C. annuum* L.) pepper (medium hot). The capsaicinoids concentrations in freeze-dried, oven-dried, and refractive window-dried samples were significantly lower than using the natural convective drying (NCD) method. The NCD is a slow drying process at ambient temperature, while the other drying processes use high temperatures (> 60 °C). The major loss of capsaicinoids in these processes can also be caused by water removal from the pepper puree. Conversely, Maurya et al. [80] reported freeze-drying was the most efficient method for retaining capsaicin content over other drying methods (e.g., sun drying, hot air oven drying, microwave-vacuum drying), although it resulted in *C. annuum* samples with lower water activity. According to the authors [80], before the drying processes, all samples of *C. annuum* had previously undergone blanching using hot water at 90 °C. The boiling process negatively influenced capsaicinoid content and other phytochemical components, which may alter their bioaccessibility and bioavailability in *Capsicum* samples [81,82].

In the smoking process, pepper fruits are dried using smoke from burning firewood. Moreno-Escamilla et al. [83] reported that pecan–oak smoked jalapeño pepper samples (221.13 ± 55.64) showed a total capsaicinoid concentration lower than fresh pepper (1302.50 ± 359.02), oak–poplar smoked jalapeño samples (1234.93 ± 265.07), and pecan wood smoked jalapeño samples (834.61 ± 72). The authors determined that the type of wood used in the smoking process influenced the *Capsicum* phytochemical content. The reduction of capsaicinoids could be related to high temperatures throughout the smoking process, which can catalyse fragmentation and oxidation of capsaicin to produce vanillin [84]. However, the decrease in capsaicinoids is not a unanimous result of the smoking process. The ripening level of pepper pods used, adjacent processing (e.g., pickling, blanched), and smoking/drying processes features of each locality can explain the differences in capsaicinoid accumulation after these processes [80,81].

In drought-stressed plants, their growth and secondary metabolite production can be significantly affected [33]. Previous studies have reported an increase in capsaicinoid content under a water deficit [61,68]. However, depending on the level of drought stress and plant stage development, it can lead to a considerable decrease in capsaicinoid content [61,63,85]. Jeeatid et al. [61] evaluated the influence of water stress on capsaicinoid accumulation in different pepper cultivars of *C. chinense* (e.g., Bhut Jolokia, Akanee Pirote, Orange Habanero, and BGH1719). The authors observed different pungency levels after flowering and reported that severe drought stress reduced or restricted the increase in capsaicinoid content in all analysed cultivars. Furthermore, under drought stress, high-pungency cultivars with large fruits were more susceptible to changes in total capsaicinoid content than smaller fruit cultivars [85,86].

During storage, total capsaicinoid concentrations are mainly affected by temperature and storage time. Giuffrida et al. [87] reported that, during long-term storage, total capsaicinoids decreased about 75% at room temperature (20 to 24 °C) and were stable at low temperatures (<20 °C). Decreased capsaicinoids were not homogeneous over 12 months of storage at room temperature; capsaicin and dihydrocapsaicin content decreased, while nordihydrocapsaicin was almost unchanged, and only homocapsaicin significantly increased (range from 2.5 to 3.5% of total capsaicinoids) [87]. This suggests that transformation and conversion between capsaicinoid isomeric forms are possible [87,88].

Furthermore, different packaging materials (polyethene and jute bags) can affect capsaicinoid levels in the final *Capsicum* product [89,90]. Iqbal et al. [89] showed that in polyethylene-packed red peppers, capsaicin and dihydrocapsaicin decreased in a range c.a. 9 to 13% and 11 to 15%, respectively. For red peppers stored in jute bags, the reduction percentage of capsaicin and dihydrocapsaicin ranged from 9 to 14% and 10 to 16%, respectively. To build on these results and form a more definitive conclusion, more studies on other types of packaging and different *Capsicum* products are needed. Currently, there is no information about the influence of storage parameters on capsinoid content.

To reduce the growth of unwanted spoilage microorganisms on *Capsicum* pepper and its derivatives, gamma irradiation has been used [91,92]. The effect of gamma irradiation on both capsaicinoid biosynthesis and chemical stability has been a controversial topic. Previous studies have reported that low gamma irradiation doses may significantly increase [88,91,93,94] or not change capsaicinoid content concentrations [90,95]. Iqbal et al. [90] reported that gamma irradiation at 2, 4, or 6 kGy did not affect the capsaicin and dihydrocapsaicin content in *C. annuum.* In contrast, Kyung et al. [94] reported that the total capsaicinoids content (e.g., capsaicin, dihydrocapsaicin, and nordihydrocapsaicin) was higher (c.a. 59%) in red pepper samples treated with a low dose rates of gamma irradiation (c.a. 106 mg/100 g; 1.8 kGy/h), than in non-irradiated pepper samples (c.a. 67 mg/100 g) or those irradiated at high doses (c.a. 99 mg/100 g; 9 kGy/h). These results seem to indicate that gamma irradiation can be safely used at a low rate to promote capsaicinoid stability in pepper fruits. On the other hand, the increase in gamma irradiation shows a negative effect on capsaicinoid stability [96,97]. Duah et al. [93] reported that pepper samples irradiated at 10 kGy decreased c.a. 12 to 25% of capsaicinoid content when compared to non-irradiated samples. The authors determined that, as the irradiation dose increased, the total capsaicinoid content decreased in red pepper. Meanwhile, some studies have reported slight changes and increases in capsaicin, dihydrocapsaicin, and homodihydrocapsaicin contents when high doses of gamma irradiation (≥ 10 kGy) are supplied [88,93,98]. Capsaicinoids content has been treated with other types of irradiations (e.g., electron beams and X-rays) at doses up to 10 kGy with no significant changes in capsaicin or dihydrocapsaicin contents between the irradiated and control samples [98,99]. Overall, the degree of synthesis and degradation of capsaicinoids is beam dose-dependent, but is also strongly affected by cultivar, growth, and storage conditions (Figure 2) [88,93].

## 5. Capsaicinoids Antifungal Activity

The antifungal capability of capsaicinoids has been evaluated in vivo, in vitro, in post-harvest tests, and as a component of nanomaterials. As mentioned above, all capsaicinoids have a similar structure (e.g., vanillyl ring, an amide linkage, and a hydrophobic tail), varying only by the length of the aliphatic side chain (ranges from 9 to 11 °C), and the presence or absence of unsaturation (Figure 1).

The antifungal activity of capsaicinoids has been attributed to the existence of the polar moiety (hydroxyl group of the vanillyl ring), and mainly to the lipophilic part of their chemical structure (e.g., acyl chain) [100]. It has also been suggested that the side chain has more inhibitory activity than the phenolic part of the molecule. Indeed, the number of carbons and double bonds present in the capsaicinoid side chain can affect its interaction with the fungal lipid bilayers [32,100,101]. This indicates that the capsaicinoids’ antifungal mechanisms involve osmotic stress and damage to the plasma membrane structure [102].

In *Capsicum* plants, capsaicinoids can act by triggering the pathways of antifungal defences. Capsaicinoids have been reported to be able to induce resistance against plant pathogens, such as *Verticillium dahlia* [100]. This indirect inhibition of fungal growth can include the enhancer expression of host defence genes and chitinase activity [100,103].

Despite their ability to produce capsaicinoids, *Capsicum* plants are susceptible to pathogens such as *Fusarium oxysporum*, *Colletotrichum capsica*, and *Botrytis cinerea* [58,104,105]. Some studies have established a correlation between the pungency index of the *Capsicum* plant and resistance to fungal pathogens [32,106]; while other studies did not detect such a correlation between pungency and fungal control, nor a consistently higher susceptibility of non-pungent peppers to spoilage fungi [107,108,109].

Further to the biotic factors and the plants’ own physiology ability to respond to pathogenic invaders, it is pivotal to highlight the fungal physiology plasticity. Alternative respiratory enzymes and capsaicin degradation are mechanisms that can be used by pathogenic fungi to overcome the stressful environment imposed by the presence of capsaicinoids [32]. In non-pungent peppers, despite the low concentration of capsaicin and capsaicinoids analogues, other phytoalexins (e.g., carotenoids, flavonoids, lactones, terpenes) can act as part of the plant defence system [30].

Most antifungal activity has been tested with extracts of *C. annuum*, *C. chinense*, and *C. frutescens*, showing promising antifungal potential, but with variable inhibition results (Table 1). The extracts are obtained from different varieties and parts of *Capsicum* (e.g., seed, pericarp, and whole plant) and extracted with different approaches, which renders an accurate comparison more difficult. Pure and synthetic substances have shown promising results, although the costs of the products are an unfavourable factor. Fungicidal and fungistatic activities of *Capsicum* extracts and purified capsaicinoids have been demonstrated in vitro. The growth rate of several pathogenic fungi of *Capsicum* plants (*Botrytis cinerea*, *Cladosporium cucumerinum*, *Colletotrichum gloeosporioides*, *Rhizoctonia solani*), spoilage fungi of agro-products (*Aspergillus flavus*, *A. niger, Fusarium oxysporum, Fusarium* sp., *Penicillium digitatum, P. expansum*), and mycotoxigenic fungal strains (*Aspergillus parasiticus*, *Aspergillus* section *Nigri*) has been reduced in the presence of capsaicinoid compounds (Table 1).

According to the results obtained in our research group via the research project ANID/FONDECYT/1221024 (*unpublished data*), fungal strains belonging to the genera *Aspergillus*, *Fusarium*, *Penicillium*, and *Rhizopus* presented macro- and micro-morphological modifications after treatment with pepper pod extracts obtained from the different varieties of *Capsicum* spp. Even for some fungal genera, after treatment, the isolates were not able to produce conidiophores. The results obtained indicate the fungistatic potential of the assessed pepper pod extracts. In fact, the pepper pod extracts can act by controlling fungal growth and reproduction. This is an important characteristic of the assessed pepper pod extracts once they do not kill fungal biodiversity, but could control their growth, reproduction, and mycotoxin production in the field.

The anti-mycotoxigenic potential of capsaicinoids has also been evaluated. Kollia et al. [110] investigated the anti-ochratoxigenic and antifungal activity of capsaicin against *Aspergillus carbonarius* and four other strains of *Aspergillus* section *Nigri*. The authors reported that capsaicin effectively restricted ochratoxin A (OTA) production, and that the growth of all *Aspergillus* strains was suppressed by over 50%.

Buitimea-Cantúa et al. [111] analysed the anti-aflatoxigenic and antifungal activity of *C. chinense* fruit extract and synthetic capsaicin. Both presented antifungal and anti-aflatoxigenic activity against *A. parasiticus*, yet synthetic capsaicin analogue was more efficient. These results suggest that capsaicin and *Capsicum* extract influence aflatoxins biosynthesis either by downregulating genes or by inhibiting fungal growth. The capsaicinoids’ modulatory effect on other mycotoxins should be further explored.

Furthermore, the exogenous application of capsaicinoids has been tested on apples (fruit), avocados (fruit), tomatoes (plant and fruit), and peppers (plant and fruit) [100,106,112,113,114]. Zanotto [113] evaluated, through in vitro and post-harvest tests (directly on apples), the antifungal activity of capsaicin and five synthetic capsaicin analogues (CAP-1, CAP-2, CAP-3, CAP-4, and CAP-5) against *C. gloeosporioides* and *P. expansum*. Among capsaicin analogues, the best results obtained from the in vitro tests were CAP-3 and CAP-4, with minimum inhibitory concentrations (MIC) of 600 µM against *Penicillium expansum*, while for *C. gloeosporioides* all analysed capsaicin analogues showed a MIC of 800 µM. In the tests carried out directly on apples, CAP-3 and CAP-4 had no fungicidal action, yet both retarded the growth of *P. expansum*, showing a fungistatic effect [113]. Similarly, Valencia-Hernandez [112] analysed the antifungal activity of the synthetic capsaicinoid oleoresin (CO) containing 70% nonivamide and 30% dihydrocapsaicin on tomato fruits after harvest. According to the authors, treatment with CO 0.05% and CO 0.20% reduced weight loss and fruit damage compared to the control [112].

Until now, the main data on the antifungal potential of capsinoids and their analogues has been mostly obtained from in vitro tests. Overall, fruit extracts and pure or synthesised analogue substances have shown positive results; though the percentages of inhibitory action have varied depending on the fungal species and strain antagonised. Furthermore, in most cases, the inhibition of fungal growth has been dose-dependent, which means that substances with higher concentrations are needed for greater effectiveness.

To the best of our knowledge, there have been very few studies that have applied capsaicinoids to plants in the field [112,115,116]. García [115] analysed the in vivo properties of vanillyl nonanoate (VNT), a synthetic capsinoid analogue, to control both *Phytophthora capsica* (oomycete) and *B. cinerea*. According to the authors, VNT treatment reduced the percentage of expanding lesions caused by *P. capsica* and *B. cinerea* in *C. annum* plants by around 25% and 30%, respectively. Valencia-Hernandez [112] analysed the antimicrobial activity of a synthetic capsaicinoid oleoresin (CO, containing 70% nonivamide and 30% dihydrocapsaicin) against *F. oxysporum* inoculates in the soil of tomato seedlings. The damage severity caused by *F. oxysporum* slightly decreased after CO foliar applications at 0.05 and 0.10%. Vázquez-Fuentes [116] conducted in vivo experiments to evaluate the suitability of avocado and tomato plants for treatment with the synthetic capsaicinoid ABX-I (N-vanillyl-butanamide). Both plants were sprayed with 0, 400 μM, 800 μM, and 1600 μM ABX-I twice a week for one month. As a result, neither plant showed any phenotypic alteration compared with plants sprayed with the control solution. The ABX-I antifungal activity against *B. cinerea* and *C. gloeosporioides* was only tested in vitro.

The results of in vivo studies have indicated that the direct application of synthetic capsaicinoid analogues to the plant led to a decrease in lesion expansion caused by phytopathogens. Furthermore, when sprayed on the plant, capsaicinoid analogues did not affect plant productivity or the quality of fruits or leaves produced. Despite the promising results, there is still room for improvement. To date, all in vivo tests have only used synthetic analogue substances (e.g., ABX-I, CO, VNT). In addition to the synthetic analogue compounds, the in vivo experiments should be expanded using *Capsicum* plant extracts. These experiments should first be scaled up to greenhouse conditions, and then later validated in open field conditions. From this, it could be determined whether factors found in the field (e.g., temperature, sunlight, pH, fruit tissue features, levels of nutrients, and natural phenolic compounds) could limit or enhance the effectiveness of these compounds.

It is then important to define at which stage of the production chain capsinoids and their analogues would be best applied. Though they could have fungistatic activity and not interfere with the organoleptic properties of the agrifood product, their large-scale production, either in pure or synthesized analogue molecules, is an additional obstacle to overcome. It is feasible to suggest that these compounds would be a strategic tool to improve product conservation during the seed and post-harvest stages. Both stages are critical for fungal contamination and require a smaller applicable volume of biofungicide. Further studies are needed to confirm these hypotheses.

Finally, a new approach has emerged, associating capsaicinoids and capsinoids with natural polymers, lipids, or essential oils in nano-formulations, or linking them to fibres, pellets, films, or membranes [117,118,119,120]. The development of these kinds of materials can unravel new opportunities for their application as biofungicides and protective membranes for the long-term storage of agrifood products [117,120].

**Table 1:** In vitro analysis of antifungal activities of capsaicinoids and capsinoids as pure molecules or pepper extracts.

Plant Species	Pure Molecule (PM) or RAW Extract (RE)	Plant Substrate	Compound Name	Concentration	Fungal Taxa	**Inhibition (%)**	Reference
*Capsicum annuum*	RE	Seed	NI	5 mg mL^−1^	*Colletotrichum gloeosporioides*	46.4	[121]
*Capsicum annuum*	RE	Seed	NI	10 mg mL^−1^	*Colletotrichum gloeosporioides*	54.6	[121]
*Capsicum annuum*	RE	Seed	NI	5 mg mL^−1^	*Colletotrichum gloeosporioides*	25.0	[121]
*Capsicum annuum*	RE	Seed	NI	10 mg mL^−1^	*Colletotrichum gloeosporioides*	38.1	[121]
*Capsicum annuum*	RE	Pericarp	NI	5 mg mL^−1^	*Colletotrichum gloeosporioides*	20.7	[121]
*Capsicum annuum*	RE	Pericarp	NI	10 mg mL^−1^	*Colletotrichum gloeosporioides*	43.6	[121]
*Capsicum annuum*	RE	Pericarp	NI	5 mg mL^−1^	*Colletotrichum gloeosporioides*	21.4	[121]
*Capsicum chinense*	RE	Fruit	NI	50 mg mL^−1^	*Aspergillus parasiticus* ATCC 16992	23.5	[111]
*Capsicum chinense*	RE	Fruit	NI	75 mg mL^−1^	*Aspergillus parasiticus* ATCC 16992	39.0	[111]
*Capsicum chinense*	RE	Fruit	NI	150 mg mL^−1^	*Aspergillus parasiticus* ATCC 16992	50.0	[111]
*Capsicum chinense*	RE	Fruit	NI	250 mg mL^−1^	*Aspergillus parasiticus* ATCC 16992	65.0	[111]
*Capsicum chinense*	RE	Fruit	NI	300 mg mL^−1^	*Aspergillus parasiticus* ATCC 16992	76.0	[111]
*Capsicum chinense*	PM	Unknown	Capsaicin	50 mg mL^−1^	*Aspergillus parasiticus* ATCC 16992	50.0	[111]
*Capsicum chinense*	PM	Unknown	Capsaicin	75 mg mL^−1^	*Aspergillus parasiticus* ATCC 16992	58.0	[111]
*Capsicum chinense*	PM	Unknown	Capsaicin	150 mg mL^−1^	*Aspergillus parasiticus* ATCC 16992	60.0	[111]
*Capsicum chinense*	PM	Unknown	Capsaicin	200 mg mL^−1^	*Aspergillus parasiticus* ATCC 16992	67.0	[111]
*Capsicum chinense*	PM	Unknown	Capsaicin	250 mg mL^−1^	*Aspergillus parasiticus* ATCC 16992	77.0	[111]
*Capsicum chinense*	PM	Unknown	Capsaicin	300 mg mL^−1^	*Aspergillus parasiticus* ATCC 16992	80.0	[111]
*Capsicum frutescens*	RE	Leaf	NI	10 mg mL^−1^	*Aspergillus flavus*	88.1	[122]
*Capsicum frutescens*	RE	Leaf	NI	20 mg mL^−1^	*Aspergillus niger*	79.3	[122]
*Capsicum frutescens*	RE	Leaf	NI	5 mg mL^−1^	*Penicillium* sp.	20.5	[122]
*Capsicum frutescens*	RE	Leaf	NI	5 mg mL^−1^	*Rhizopus* sp.	69.0	[122]
*Capsicum frutescens*	RE	Leaf	NI	5 mg mL^−1^	*Aspergillus flavus*	79.2	[122]
*Capsicum frutescens*	RE	Leaf	NI	10 mg mL^−1^	*Aspergillus niger*	88.3	[122]
*Capsicum frutescens*	RE	Leaf	NI	1.25 mg mL^−1^	*Penicillium* sp.	32.9	[122]
*Capsicum frutescens*	RE	Leaf	NI	5 mg mL^−1^	*Rhizopus* sp.	77.2	[122]
*Capsicum frutescens*	RE	Whole plant	NI	3 µg mL^−1^	*Aspergillus niger*	91.4	[123]
*Capsicum frutescens*	RE	Whole plant	NI	3 µg mL^−1^	*Penicillium digitatum*	83.1	[123]
*Capsicum frutescens*	RE	Whole plant	NI	3 µg mL^−1^	*Fusarium* sp.	87.6	[123]
*Capsicum frutescens*	RE	Whole plant	NI	2 µg mL^−1^	*Aspergillus niger*	78.9	[123]
*Capsicum frutescens*	RE	Whole plant	NI	2 µg mL^−1^	*Penicillium digitatum*	74.0	[123]
*Capsicum frutescens*	RE	Whole plant	NI	2 µg mL^−1^	*Fusarium* sp.	69.3	[123]
*Capsicum frutescens*	RE	Whole plant	NI	1 µg mL^−1^	*Aspergillus niger*	73.2	[123]
*Capsicum frutescens*	RE	Whole plant	NI	1 µg mL^−1^	*Penicillium digitatum*	69.2	[123]
*Capsicum frutescens*	RE	Whole plant	NI	1 µg mL^−1^	*Fusarium* sp.	61.3	[123]
*Capsicum frutescens*	RE	Whole plant	NI	0.5 µg mL^−1^	*Aspergillus niger*	55.7	[123]
*Capsicum frutescens*	RE	Whole plant	NI	0.5 µg mL^−1^	*Penicillium digitatum*	51.5	[123]
*Capsicum frutescens*	RE	Whole plant	NI	0.5 µg mL^−1^	*Fusarium* sp.	49.0	[123]
*Capsicum* sp.	RE	Fruit	NI	500 mg mL^−1^	*Sphaeropsis sapinea*	100.0	[124]
*Capsicum* sp.	RE	Fruit	NI	500 mg mL^−1^	*Sphaeropsis sapinea*	100.0	[124]
*Capsicum* sp.	RE	Fruit	NI	350 mg mL^−1^	*Sphaeropsis sapinea*	40.0	[124]
*Capsicum* sp.	RE	Fruit	NI	350 mg mL^−1^	*Sphaeropsis sapinea*	100.0	[124]
*Capsicum* sp.	PM	Unknown	Capsaicin	25 mg mL^−1^	*Colletotrichum* truncatum	15.0	[109]
*Capsicum* sp.	PM	Unknown	Capsaicin	50 mg mL^−1^	*Colletotrichum* truncatum	35.0	[109]
*Capsicum* sp.	PM	Unknown	Capsaicin	100 mg mL^−1^	*Colletotrichum* truncatum	41.0	[109]
*Capsicum* sp.	PM	Unknown	Capsaicin	200 mg mL^−1^	*Colletotrichum* truncatum	59.0	[109]
*Capsicum* sp.	PM	Unknown	Capsaicin	122.16 µg mL^–1^	*Penicillium expansum*	75.0	[125]
*Capsicum* sp.	PM	Unknown	Capsaicin	76.4 µg mL^−1^	*Verticillium* dahliae VDL	22.0	[100]
*Capsicum* sp.	PM	Unknown	Capsaicin	76.4 µg mL^−1^	*Verticillium* dahliae UDC53Vd	35.3	[100]
*Capsicum* sp.	PM	Unknown	Capsaicin	76.4 µg mL^−1^	*Verticillium* dahliae 2694	0.9	[100]
*Capsicum* sp.	PM	Unknown	Capsaicin	76.4 µg mL^−1^	*Verticillium* dahliae 2884	19.9	[100]
*Capsicum* sp.	PM	Unknown	Capsaicin	76.4 µg mL^−1^	*Verticillium* tricorpus 2695	2.7	[100]
*Capsicum* sp.	PM	Unknown	Capsaicin	76.4 µg mL^−1^	Botrytis cinerea 2850	20.6	[100]
*Capsicum* sp.	PM	Unknown	Capsaicin	76.4 µg mL^−1^	Rhizoctonia solani 2815	32.0	[100]
*Capsicum* sp.	PM	Unknown	Capsaicin	76.4 µg mL^−1^	Fusarium oxysporum 2715	18.7	[100]
*Capsicum* sp.	PM	Unknown	Capsaicin	76.4 µg mL^−1^	Pythium ultimum 2364	29.2	[100]
*Capsicum* sp.	PM	Unknown	Capsaicin	76.4 µg mL^−1^	Phytophthora capsici P12M	25.2	[100]
*Capsicum* sp.	PM	Unknown	Capsaicin	76.4 µg mL^−1^	Phytophthora capsici P15M	44.4	[100]
*Capsicum* sp.	PM	Unknown	Capsaicin	76.4 µg mL^−1^	Phytophthora capsici UDC1Pc	40.5	[100]
*Capsicum* sp.	PM	Unknown	Capsaicin	76.4 µg mL^−1^	Phytophthora capsici UDC141Pc	46.8	[100]
*Capsicum* sp.	PM	Unknown	Capsaicin	76.4 µg mL^−1^	Phytophthora capsici UDC288Pc	24.9	[100]
*Capsicum* sp.	PM	Unknown	Capsaicin	76.4 µg mL^−1^	Phytophthora capsici UDC299Pc	73.2	[100]
*Capsicum* sp.	PM	Unknown	Capsaicin	76.4 µg mL^−1^	Phytophthora capsici UDC265Pc	59.9	[100]
*Capsicum* sp.	PM	Unknown	Capsaicin	76.4 µg mL^−1^	Phytophthora capsici UDC384Pc	55.8	[100]
*Capsicum* sp.	PM	Unknown	Capsaicin	152.7 µg mL^−1^	*Verticillium* dahliae VDL	31.7	[100]
*Capsicum* sp.	PM	Unknown	Capsaicin	152.7 µg mL^−1^	*Verticillium* dahliae UDC53Vd	46.1	[100]
*Capsicum* sp.	PM	Unknown	Capsaicin	152.7 µg mL^−1^	*Verticillium* dahliae 2694	3.7	[100]
*Capsicum* sp.	PM	Unknown	Capsaicin	152.7 µg mL^−1^	*Verticillium* dahliae 2884	29.5	[100]
*Capsicum* sp.	PM	Unknown	Capsaicin	152.7 µg mL^−1^	*Verticillium* tricorpus 2695	10.3	[100]
*Capsicum* sp.	PM	Unknown	Capsaicin	76.4 µg mL^−1^	*Botrytis cinerea* 2850	35.9	[100]
*Capsicum* sp.	PM	Unknown	Capsaicin	152.7 µg mL^−1^	Rhizoctonia solani 2815	45.6	[100]
*Capsicum* sp.	PM	Unknown	Capsaicin	152.7 µg mL^−1^	Fusarium oxysporum 2715	33.3	[100]
*Capsicum* sp.	PM	Unknown	Capsaicin	152.7 µg mL^−1^	Pythium ultimum 2364	49.1	[100]
*Capsicum* sp.	PM	Unknown	Capsaicin	152.7 µg mL^−1^	Phytophthora capsici P12M	38.7	[100]
*Capsicum* sp.	PM	Unknown	Capsaicin	152.7 µg mL^−1^	Phytophthora capsici P15M	59.5	[100]
*Capsicum* sp.	PM	Unknown	Capsaicin	152.7 µg mL^−1^	Phytophthora capsici UDC1Pc	62.4	[100]
*Capsicum* sp.	PM	Unknown	Capsaicin	152.7 µg mL^−1^	Phytophthora capsici UDC141Pc	60.6	[100]
*Capsicum* sp.	PM	Unknown	Capsaicin	152.7 µg mL^−1^	Phytophthora capsici UDC288Pc	65.5	[100]
*Capsicum* sp.	PM	Unknown	Capsaicin	152.7 µg mL^−1^	Phytophthora capsici UDC299Pc	79.1	[100]
*Capsicum* sp.	PM	Unknown	Capsaicin	152.7 µg mL^−1^	Phytophthora capsici UDC265Pc	69.6	[100]
*Capsicum* sp.	PM	Unknown	Capsaicin	152.7 µg mL^−1^	Phytophthora capsici UDC384Pc	68.3	[100]
*Capsicum* sp.	PM	Unknown	Dihydrocapsaicin	153.7 µg mL^−1^	*Verticillium dahliae*	37.4	[100]
*Capsicum* sp.	PM	Unknown	Dihydrocapsaicin	153.7 µg mL^−1^	*Verticillium dahliae*	62.6	[100]
*Capsicum* sp.	PM	Unknown	Capsaicin	60 mg mL^−1^	*Aspergillus carbonarius* ATHUM 2854	89.7	[110]
*Capsicum* sp.	PM	Unknown	Capsaicin	60 mg mL^−1^	*Aspergillus* section *Nigri*	54.5	[110]
*Capsicum* sp.	PM	Unknown	Capsaicin	60 mg mL^−1^	*Aspergillus* section *Nigri* ATHUM 6998	78.9	[110]
*Capsicum* sp.	PM	Unknown	Capsaicin	60 mg mL^−1^	*Aspergillus* section *Nigri* ATHUM 6999	79.0	[110]
*Capsicum* sp.	PM	Unknown	Capsaicin	60 mg mL^−1^	*Aspergillus* section *Nigri* ATHUM 7000	70.0	[110]
*Capsicum* sp.	PM	Unknown	ABX-I	115.6 mg mL^−1^	*Botrytis cinerea*	2.0	[116]
*Capsicum* sp.	PM	Unknown	ABX-I	231.2 mg mL^−1^	*Botrytis cinerea*	9.0	[116]
*Capsicum* sp.	PM	Unknown	ABX-I	462.4 mg mL^−1^	*Botrytis cinerea*	33.0	[116]
*Capsicum* sp.	PM	Unknown	ABX-I	115.6 mg mL^−1^	*Colletotrichum* gloeosporioides	18.0	[116]
*Capsicum* sp.	PM	Unknown	ABX-I	231.2 mg mL^−1^	*Colletotrichum* gloeosporioides	28.0	[116]
*Capsicum* sp.	PM	Unknown	ABX-I	462.4 mg mL^−1^	Colletotrichum gloeosporioides	65.0	[116]
*Capsicum* sp.	PM	Unknown	ABX-I	115.6 mg mL^−1^	*Rhizoctonia solani*	44.0	[116]
*Capsicum* sp.	PM	Unknown	ABX-I	231.2 mg mL^−1^	*Rhizoctonia solani*	62.0	[116]
*Capsicum* sp.	PM	Unknown	ABX-I	462.4 mg mL^−1^	*Rhizoctonia solani*	87.0	[116]
*Capsicum* sp.	PM	Unknown	ABX-I	115.6 mg mL^−1^	*Fusarium* sp.	41.0	[116]
*Capsicum* sp.	PM	Unknown	ABX-I	231.2 mg mL^−1^	*Fusarium* sp.	51	[116]
*Capsicum* sp.	PM	Unknown	ABX-I	462.4 mg mL^−1^	*Fusarium* sp.	67.0	[116]
*Capsicum* sp.	PM	Unknown	Capsaicin	244.3 µg mL^−1^	*Colletotrichum* gloeosporioides	60.0	[116]
*Capsicum* sp.	PM	Unknown	Capsaicin	244.3 µg mL^−1^	*Colletotrichum acutatum*	59.0	[116]

NI = Not identified.

## 6. Impact of Using Capsaicinoids and Capsinoids on Environmental and Human Health

Due to their versatile applicability, capsaicinoids and analogue molecules have received constant scientific interest [94,114,116,117,120]. Capsaicin and its analogues have been proven to exhibit antimicrobial activity against phytopathogens, such as the fungus *Botrytis cinerea*, *Aspergillus niger*, *Colletotrichum capsici, Fusarium oxysporum*, or *Rhizoctonia solani* [112,114,126,127]. Although capsaicinoids and capsinoids from pepper extracts are natural products that can be applied to control pathogenic fungi growth in plant crop production, it is important to understand the effect these compounds could have on species sharing the same environment [128]. In fact, in agricultural production, capsaicinoid molecules can play a dual role as biocidals. Firstly, the bioinsecticide potential of capsaicinoid molecules could be an eco-friendly pest management strategy to avoid the spread of phytophagous insects themselves [129]. Secondly, these compounds can also prevent fungal infection, either through their antifungal activity, but also by reducing risk, since insects can be fungal disease vectors due to their feeding mechanisms [106].

Capsaicin has a broad spectrum of repellent and insecticide activity against many species, such as stored product beetles, rice grain insects, alfalfa weevils, and some whitefly and cabbage moths [129,130,131]. The secondary metabolites produced by *Capsicum* plants, including capsaicinoids and capsinoids, have been reported to have the ability to affect insects at the cellular, tissue and organism levels [132]. For instance, low concentrations of capsaicin (10^−7^ and 10^−4^ M) were applied in *Tenebrio molitor* L. and revealed that both concentrations induced changes in its thermoregulation behaviour that ultimately affected all the physiological processes [133]. Edelson et al. [134] demonstrated that capsaicin extracts alone were only able to produce low levels of mortality in peach aphids (*Myzus persicae* Sulzer) but synergistically acted in mixtures with other insecticides, providing levels of mortality even higher than expected [134]. Li et al. [130] applied a solution with 0.05% of capsaicinoids (c.a. 96%, including c.a. 60% of CAP and c.a. 30% of DHC) against 14 agricultural insect species in laboratory and field conditions. Based on obtained results, a high insecticidal activity of capsaicinoids solution when used to control *Aphis gossypii* in *Cucumis sativus* was observed (Lethal Concentration 50—LC50 152.82 mg/L). However, a low insecticidal activity when used against *Ectropis obliquahypulina* and *Pieris rapae* was obtained (LC50 1557 and 1502 mg/L, respectively). Furthermore, results from field experiments showed that the insects’ control effect of successively spraying the capsaicinoids solution two times was significantly higher than the effect of spraying once [130].

More importantly, the application of capsaicinoids and their analogues as pesticides, unlike synthetic compounds, has very low toxicity to non-target organisms [130,135]. Up to now, there is no information on the effect of these compounds on the bee-like beneficial insect. As a natural compound, capsaicinoids and their analogues are much less hazardous than chemical pesticides. They present a low risk to humans and domestic animals and could be applied in different stages of agricultural production without risking workers [135]. Furthermore, as they are not recalcitrant molecules, they do not pose a risk of soil and groundwater contamination.

Regarding the role of capsaicin in human health, a large body of evidence about its benefits has been established [136]. The research carried out so far has shown that capsaicinoids, particularly capsaicin, have a diversity of biological functions, highlighting their roles as antioxidants, stimulants of the energetic metabolism, fat accumulating suppressors, anti-inflammatories, neurostimulants and apoptosis-alleviating agents in neurodegenerative disorders [137]. Capsaicin has demonstrated a slight analgesic action in chronic pain complaints, where topical usage promotes desensitisation to pain after recurrent applications [138]. A significant pain reduction of 57 and 33% was reported by topical use of a 0.025% capsaicin cream for treating osteoarthritis and rheumatoid arthritis in patients, respectively, which confirms the positive analgesic effects of capsaicin [139]. In addition, capsaicin has exhibited protective properties against many mutagenic and tumour-causing cells, specifically inducing apoptosis in these cells [140]. Previous studies have shown that the administration of low doses of capsaicin can suppress the growth of many human cancers, and there are even reports that high doses of capsaicin have been used to treat cancers [141]. Furthermore, it has been described that capsaicin effectively lowered the expression and activity of many proteins associated with cell cycle progression, thereby reducing the rates of proliferation and migration of the cancer cells [142].

Most applications described in this section rely on the use of pure capsaicinoids and capsinoids extracts. Although new extraction and purification protocols are continuously being reported [143], in order to meet future capsaicinoids and capsinoids demands, it will be essential to develop new synthetic capsaicinoids’ analogues. Such solutions will allow the exploitation of full potential of these compounds, while simultaneously mitigating the potential impact that the necessary large-scale production of *Capsicum* could have, particularly in terms of required soil area and associated economic costs.

New synthesis solutions that are currently being explored include approaches relying on marker-assisted selection in breeding programmes [144,145], or the use of transformed cell factories such as *Saccharomyces cerevisiae* or *Escherichia coli* [146,147,148,149]. In recent years, particularly after the release of the first hot pepper genome assembly (Mexican landrace of *Capsicum annuum* cv. CM334) [150], genetic engineering of *Capsicum* plants has become an attractive approach to transform and improve this crop of social and economic importance [151,152]. Genome editing of *Capsicum* plants is being developed through the establishment of methods for reagents delivery, such as *Agrobacterium*-mediated transformation [153], coupled with the identification of CRISPR-based tools and editing sites [154,155] and the design of feasible regeneration techniques [156]. These techniques have not yet been specifically applied to improve capsaicinoids and capsinoids content in *Capsicum* plants. However, possible targets include regulatory genes involved in the biosynthetic pathways of these compounds, which can be identified, for example, through quantitative trait locus (QTL) mapping [48,145].

## 7. Conclusions

Based on the information described and summarised in this work, the antifungal properties of capsaicinoids and capsinoids can be of value as part of the One Health concept. Overall, the use of natural compounds, such as capsaicinoids and their analogues, as biofungicides has the potential of improving food safety, nutritional value, and of overcoming antimicrobial resistance, with a lower associated risk than that of chemical fungicides and pesticides.

Capsaicinoids and capsinoids can be used in the different stages of the agricultural and food production chains with negligible risk to workers, consumers, and the surrounding environment. The beneficial characteristics of these molecules include the demonstrated fungicidal and fungistatic activities of pure *Capsicum* extracts and purified capsaicinoids. In the field production stage, capsaicinoids and their analogues can be used to control pathogenic fungi growth in plant crops and as eco-friendly alternatives to pest management.

Unlike their synthetic counterparts, such natural-based fungicides and pesticides have low toxicity to non-target organisms. Nevertheless, future studies on the effect of these compounds on bee-like beneficial insects are still necessary. In the post-harvest production stage, capsinoid analogues have the advantage of not interfering with the products’ organoleptic properties, which can assist in the conservation and long-term storage of agrifood products. Future research focusing on the development of new preservatives and packaging materials based on the use of capsaicinoids and capsinoids represents a topic of interest.

## Data Availability

Not applicable.
